# Supplementary material for: Medication safety with oral antitumour therapeutics in paediatrics (youngAMBORA): A mixed-methods approach towards a tailored care program
Source: PLoS One. 2024 Dec 5;19(12):e0315077. doi: 10.1371/journal.pone.0315077 (PMC11620591; doi:10.1371/journal.pone.0315077)
Supplement: S1 File — (DOCX) [file pone.0315077.s001.docx]

**Supporting information**

**Medication safety with oral antitumour therapeutics in paediatrics (youngAMBORA): A mixed-methods approach towards a tailored care program**

[SUPPORTING METHODS 3](#_Toc165456806)

[1. The AMBORA intervention 3](#_Toc165456807)

[SUPPORTING RESULTS 4](#_Toc165456808)

[2. Qualitative data collection 4](#_Toc165456809)

[2.1. Section A ‘Administration and handling of oral drugs’ 4](#_Toc165456810)

[S1 Table. Patients. 4](#_Toc165456811)

[S2 Table. Caregivers. 6](#_Toc165456812)

[S3 Table. Healthcare professionals. 8](#_Toc165456813)

[2.2. Section B ‘Oral medication education’ 10](#_Toc165456814)

[S4 Table. Patients. 10](#_Toc165456815)

[S5 Table. Caregivers. 11](#_Toc165456816)

[S6 Table. Healthcare professionals. 13](#_Toc165456817)

[2.3. Section C ‘Side effects’ 15](#_Toc165456818)

[S7 Table. Relevance of side effects according to patients, caregivers, and healthcare professionals. 15](#_Toc165456819)

[S8 Table. Caregivers. 18](#_Toc165456820)

[S9 Table. Healthcare professionals. 19](#_Toc165456821)

[3. Adaptation of the AMBORA intervention 20](#_Toc165456822)

[S1 Fig. Drug fact sheet – Mercaptopurine. 22](#_Toc165456823)

[S10 Table. Overview of the provided information material in the AMBORA trial [1] and in the youngAMBORA care program. 24](#_Toc165456824)

[4. Designed information material 25](#_Toc165456825)

[S11 Table. OAT fact sheets. 25](#_Toc165456826)

[S12 Table. Information brochures about side effect prevention and management. 25](#_Toc165456827)

[5. References 26](#_Toc165456828)

SUPPORTING METHODS

1. The AMBORA intervention

In brief, the AMBORA care program for adults included patient consultations at OAT initiation (week 0), and predefined follow-up sessions after one, four, and twelve weeks. The intervention addressed four key elements: counselling and training, side effect prevention and management, adherence counselling, and medication management and was supported by written standardised information material (drug fact sheets, information brochures about common side effects, and drug intake plans). Patients from the age of 18 treated with any ‘new OAT’, defined by its approval date in 2001 or later, were included in the AMBORA study [1].

SUPPORTING RESULTS

1. Qualitative data collection
   1. Section A ‘Administration and handling of oral drugs’

S1 Table. Patients.

| Survey question | Patients (N=22) |
| --- | --- |
| **Administration and handling of oral drugs** | **No of responses (%)** |
| Who makes sure that you take your medication regularly at home?  My mother and my father  My mother  My father  I do that myself  My sibling(s)  Somebody else | **22 (100.0)**  11 (50.0)  9 (40.9)  2 (9.1)  2 (9.1)  -  - |
| Who collects your medication from the pharmacy for you?  My mother and my father  My mother  Somebody else  I do that myself  My sibling(s)  My father | **22 (100.0)**  13 (59.1)  8 (36.4)  2 (9.1)  1 (4.5)  1(4.5)  - |
| Does taking your medication bother you?  Yes, that bothers me  Sometimes yes, sometimes no  No, that doesn`t bother me | **22 (100.0)**  4 (18.2)  15 (68.2)  3 (13.6) |
| Which dosage form do you think is most suitable at the time of data collection?  Tablet  Solution/drop  Capsule  Syrup  Emulsion/suspension  Powder/granulate | **21 (100.0)**  13 (61.9)  7 (33.3)  5 (23.8)  3 (14.3)  3 (14.3)  1 (4.8) |
| Do you find it difficult to swallow your medication?  Yes, I find it difficult  Sometimes yes, sometimes no  No, I don‘t find it difficult | **22 (100.0)**  4 (18.2)  6 (27.3)  12 (54.5) |
| Are you afraid of taking oral drugs?  Yes, I am afraid  Sometimes yes, sometimes no  No, I am not afraid | **22 (100.0)**  1 (4.5)  4 (18.2)  17 (77.3) |
| What might you be afraid of when swallowing oral drugs?  Taste of the drug  Size of the drug  Nausea  Other  That the drug doesn’t fit down my throat  That I can’t tolerate the medication  I don’t know  Pain | **19 (100.0)**  9 (47.4)  7 (36.8)  8 (42.1)  3 (15.8)  3 (15.8)  3 (15.8)  3 (15.8)  - |
| What probably helped you swallowing tablets or capsules?  Swallowing aids (e.g., tablet coatings)  Admixture of food  Crushing or splitting the drugs  Swallowing exercises  Reward after intake  Rituals  My family and friends  Other | **17 (100.0)**  7 (41.2)  4 (23.5)  4 (23.5)  3 (17.6)  2 (11.8)  2 (11.8)  1 (5.9)  - |
| Do you find it difficult to remember to take oral drugs regularly (adherence)?  Yes, I find it difficult  Sometimes yes, sometimes no  No, I don’t find it difficult | **21 (100.0)**  4 (19.0)  7 (33.3)  10 (47.6) |
| What tool helped you to remember to take oral drugs regularly?  My family and friends  Intake plan  Fixed integration into daily routine  I don’t know  Reminder system (e.g., alarm clock)  Mobile application  Other | **22 (100.0)**  20 (90.9)  4 (18.2)  4 (18.2)  2 (9.1)  1 (4.5)  1 (4.5)  - |

S2 Table. Caregivers.

| Survey question | Caregivers (N=44) |
| --- | --- |
| **Administration and handling of oral drugs** | **No of responses (%)** |
| How often did your child take oral drugs before cancer diagnosis?  Very frequently (> 1/week)  Frequently (> 1/month)  Occasionally (>1/6 months)  Rarely (> 1/year)  Never before | **43 (100.0)**  3 (7.0)  -  7 (16.3)  17 (39.5)  16 (37.2) |
| As a caregiver of a child diagnosed with cancer, how often do you personally take oral drugs?  Very frequently (> 1/week)  Frequently (> 1/month)  Occasionally (>1/6 months)  Rarely (> 1/year)  Never before | **41 (100.0)**  10 (24.4)  7 (17.1)  13 (31.7)  6 (14.6)  5 (12.2) |
| As a caregiver of a child diagnosed with cancer, what are your personal experiences with taking oral drugs?  Exclusively positive  Mostly positive  Neutral  Mostly negative  Exclusively negative | **41 (100.0)**  4 (9.8)  17 (41.5)  19 (46.3)  1 (2.4)  - |
| Which dosage form do you think is most suitable for your child at start of treatment?  Syrup  Solution/drop  Emulsion/suspension  Tablet  Capsule  Powder/granulate | **42 (100.0)**  27 (64.3)  21 (50.0)  13 (31.0)  10 (23.8)  4 (9.5)  1 (2.4) |
| Which dosage form do you think is most suitable for your child at the time of data collection?  Tablet  Syrup  Capsule  Solution/drop  Emulsion/suspension  Powder/granulate | **43 (100.0)**  28 (65.1)  16 (37.2)  13 (30.2)  12 (27.9)  8 (18.6)  - |
| From your point of view, what are the most challenging difficulties associated with oral drugs in peadiatrics?  Smell/taste  Swallowing problems  Refusal of intake  Adherence  Side effects  Procurement  Lack of suitable dosages  Interactions  Alternate dosage formulations  Storage | **42 (100.0)**  23 (54.8)  21 (50.0)  16 (38.1)  15 (35.7)  10 (23.8)  7 (16.7)  5 (11.9)  5 (11.9)  2 (4.8)  1 (2.4) |
| Did your child have difficulties swallowing tablets or capsules at start of treatment?  Yes, definitely  Yes, partially  Neutral  Rather not  Not at all | **44 (100.0)**  24 (54.5)  8 (18.2)  2 (4.5)  6 (13.6)  4 (9.1) |
| Does your child have difficulties swallowing tablets or capsules at time of data collection?  Yes, definitely  Yes, partially  Neutral  Rather not  Not at all | **44 (100.0)**  5 (11.4)  8 (18.2)  2 (4.5)  11 (25.0)  18 (40.9) |
| What helped your child swallowing tablets or capsules?  Crushing or splitting the drug  Swallowing aids (e.g., tablet coatings)  Admixture of food  Age-appropriate education  Reward after intake  Rituals  Swallowing exercises | **38 (100.0)**  16 (42.1)  14 (36.8)  11 (28.9)  10 (26.3)  10 (26.3)  7 (18.4)  4 (10.5) |
| Did your child refuse to take oral drugs in the past?  Yes, definitely  Yes, partially  Neutral  Rather not  Not at all | **43 (100.0)**  9 (20.9)  13 (30.2)  4 (9.3)  5 (11.6)  12 (27.9) |
| Do you/your child find it difficult to remember to take oral drugs regularly (adherence)?  Yes, definitely  Yes, partially  Neutral  Rather not  Not at all | **44 (100.0)**  3 (6.8)  4 (9.1)  4 (9.1)  20 (45.5)  13 (29.5) |
| What tools do you/your child use to remember to take oral drugs regularly?  Fixed integration into daily routine  Reminder system (e.g., alarm clock)  Intake plan  Mobile application | **33 (100.0)**  21 (63.6)  19 (57.6)  12 (36.4)  4 (12.1) |
| Do you think the medication plan, that you received for your child on discharge from hospital is clear?  Yes, definitely  Yes, partially  Neutral  Rather not  Not at all | **44 (100.0)**  20 (45.5)  18 (40.9)  2 (4.5)  4 (9.1)  - |
| If not, please describe difficulties with the medication plan and possible suggestions for improvement for a clearer design:   - ‘Different intake plans for different dosage forms.’ - ‘Specification of the volume to be administered (liquids), not just the dosage in mg or g.’ - ‚No handwritten changes.‘ - ‚Addition of indications.‘ - ‘Addition of interactions, instructions for use and side effects.’ - ‘Plan for dose reduction is not clear.’ - ‘Addition of information about the delivery capability in the pharmacy and oral explanation of the medication plan.’ - ‘Independent plan for 6-mercaptopurine would be useful.’ - ‘The medication plan is unclear. Addition of the number of tablets/capsules to be taken and not just the total dosage. Addition of information about the time of intake.‘ - ‘The indicated time of intake is unclear.’ - ‘We modified the plan, so that it suits to us.’ - ‘Specification of the volume to be administered (liquids) and addition of information about the measurement options.’ | |

S3 Table. Healthcare professionals.

| Survey question | Healthcare professionals (N=36) | | | | | |
| --- | --- | --- | --- | --- | --- | --- |
| **Administration and handling of oral drugs** | **No of responses (%)** | | | | | |
| Which of the following dosage forms do you consider most suitable for the specified age group?  Solution/drop  Emulsion/suspension  Syrup  Powder/granulate  Tablet  Capsule  Effervescent drug  Chewable tablet | <0d  32 (100.0) | 0-28d  36 (100.0) | 1-23m  36 (100.0) | 2-5y  36 (100.0) | 6-11y  36 (100.0) | 12-18y  36  (100.0) |
|  | 30  (93.8)  15 (46.9)  6 (18.8)  2 (6.3)  -  -  -  - | 33 (91.7)  22 (61.1)  15 (41.7)  2 (5.6)  -  -  -  - | 24 (66.7)  32 (88.9)  33 (91.7)  7 (19.4)  1 (2.8)  -  -  2 (5.6) | 22 (61.1)  33 (91.7)  31 (86.1)  16 (44.4)  7 (19.4)  1 (2.8)  7 (19.4)  9 (25.0) | 16 (44.4)  20 (55.6)  22 (61.1)  17 (47.2)  27 (75.0)  11 (30.6)  14 (38.9)  20 (55.6) | 12 (33.3)  4 (11.1)  5 (13.9)  14 (38.9)  35 (97.2)  31 (86.1)  14 (38.9)  21 (58.3) |
| From your point of view, what are the most challenging difficulties associated with oral drugs in paediatrics?  Smell/taste  Refusal of intake  Swallowing problems  Lack of suitable dosages  Procurement  Alternate dosage formulations  Adherence  Interactions  Storage  Side effects | **36 (100.0)**  32 (88.9)  31 (86.1)  28 (77.8)  21 (58.3)  8 (22.2)  6 (16.7)  4 (11.1)  3 (8.3)  1 (2.8)  - | | | | | |
| From your experience, what can help children swallowing tablets or capsules?  Swallowing aids (e.g., tablet coatings)  Admixture of food  Crushing or splitting the drugs  Introducing rituals  Age-appropriate education  Reward after intake  Swallowing exercises | **36 (100.0)**  30 (83.3)  28 (77.8)  25 (69.4)  23 (63.9)  20 (55.6)  18 (50.0)  7 (19.4) | | | | | |
| From your experience, what tools can help children to remember to take oral drugs regularly?  Intake plan  Fixed integration into daily routine  Mobile Application  Reminder system (e.g., alarm clock) | **36 (100.0)**  27 (75.0)  27 (75.0)  22 (61.1)  15 (41.7) | | | | | |
| Do you think the medication plan you provide your patients on discharge from hospital is clear?  Yes, definitely  Yes, partially  Neutral  Rather not  Not at all | **35 (100.0)**  3 (8.6)  18 (51.4)  2 (5.7)  9 (25.7)  3 (8.6) | | | | | |

| If not, please describe difficulties with the medication plan and possible suggestions for improvement for a clearer design:   - ‘Too much unnecessary information and confusing information on the duration of intake.’ - ‚Highlight intake time better.‘ - ‘Inserting the clock time is not useful. Transfer of medication from the electronic prescription software to the medication plan is often not correct. Additional plans are required for some drugs (e.g., dexamethasone, mercaptopurine).’ - ‘Drugs should be labeled more clearly. Addition of the number of tablets/capsules to be taken and not just the total dosage.’ - ‘The presentation must be simpler.’ - ‘The design is bad and unclear. Time of intake with clock times is confusing.’ - ‘Indication of dose strength is confusing.’ - ‘Ambiguities concerning the units (mg or ml) and not clear which dosage für which drug.’ - ‘Unclear for reduction schemes (e.g., cortisone).’ - ‘Dose and dosage can’t be distinguished, this can lead to confusion.’ - ‚Not clear enough.‘ - ‘Time of intake and dosage form are not clear.’ - ‘Not clear and difficult to understand.’ - ‘Often only the active ingredients of the medications are known.’ - ‘No distinction between dose and dosage.’ - ‘Too confusing. Mixing of units. Always state the dose in mg if possible.’ - ‘Not clear and no subsequent change possible.’ - ‘Indication of alternating intake regimens is confusing.’ |
| --- |

- 1. Section B ‘Oral medication education’

S4 Table. Patients.

| Survey question | Patients (N=22) |
| --- | --- |
| **Oral medication education** | **No. Responses (%)** |
| Would you liked to be involved in oral medication education?  Yes  A little bit  No | **22 (100.0)**  9 (40.9)  9 (40.9)  4 (18.2) |

S5 Table. Caregivers.

| Survey question | Caregivers (N= 44) |
| --- | --- |
| **Oral medication education** | **No of responses (%)** |
| Do you consider the information you received at the time of a new prescription of an oral drug to be sufficient?  Yes, definitely  Yes, partially  Neutral  Rather not  Not at all | **43 (100.0)**  16 (37.2)  16 (37.2)  2 (4.7)  8 (18.6)  1 (2.3) |
| In which subject would you have liked to be more informed about oral drugs?  Prevention of side effects  Handling of side effects  Complementary therapy  Mode of action  Interactions  Application instructions  Indication  Safety instructions (OAT)  Storage  Change of dosage form  Procurement | **26 (100.0)**  15 (57.7)  15 (57.7)  11 (42.3)  10 (38.5)  9 (34.6)  9 (34.6)  8 (30.8)  5 (19.2)  4 (15.4)  2 (7.7)  1 (3.8) |
| How do you currently obtain information about oral drugs?  Treatment team  Package leaflet  Pharmacy  Internet  Other patients and caregivers  Acquaintances  Self-help groups | **42 (100.0)**  30 (71.4)  22 (52.4)  16 (38.1)  15 (35.7)  5 (11.9)  1 (2.4)  - |
| Do you consider a clinical pharmacological/pharmaceutical consultation on the use of oral drugs to be useful?  Yes, definitely  Yes, partially  Neutral  Rather not  Not at all | **43 (100.0)**  20 (46.5)  15 (34.9)  3 (7.0)  4 (9.3)  1 (2.3) |
| If not, please describe why you do not consider a clinical pharmacological/pharmaceutical consultation on the use of oral drugs to be useful:   - ‘I trust the doctor’s statement.’ - ‘In my opinion, the treatment team has more experience.’ - ‘Only makes sense if the pharmacist is familiar with the subject and not just reading up on it.’ - ‘Dosages are calculated differently by the pharmacist (e.g., cotrimoxazole).’ | |
| At what point in the therapy do you consider a pharmacological/ pharmaceutical consultation on the use of oral drugs to be useful?  At time of every new prescription of an oral drug  Before a therapy block  At time of hospital discharge  At time of first oncological diagnosis  As needed | **37 (100.0)**  27 (73.0)  15 (40.5)  9 (24.3)  6 (16.2)  4 (10.8) |

| How would you grade the importance of the following contents within a pharmacological/pharmaceutical consultation?  Prevention of side effects  Time of intake  Swallowing problems  Application instructions  Adherence  Handling of side effects  Interactions  Mode of action  Storage  Duration of intake  Complementary therapy  Procurement  Indication | Very important | Important | Less important | Total |
| --- | --- | --- | --- | --- |
|  | 36 (92.3)  29 (76.3)  24 (63.2)  28 (73.7)  19 (55.9)  37 (92.5)  29 (76.3)  27 (69.2)  23 (59.0)  25 (67.6)  22 (56.4)  20 (52.6)  27 (75.0) | 0 (0.0)  8 (21.1)  8 (21.1)  5 (13.2)  8 (23.5)  1 (2.5)  6 (15.8)  10 (25.6)  10 (25.6)  8 (21.6)  6 (15.4)  9 (23.7)  4 (11.1) | 3 (7.7)  1 (2.6)  6 (15.8)  5 (13.2)  7 (20.6)  2 (5.0)  3 (7.9)  2 (5.1)  5 (12.8)  4 (10.8)  11 (28.2)  9 (23.7)  5 (13.9) | 39 (100.0)  38 (100.0)  38 (100.0)  38 (100.0)  34 (100.0)  40 (100.0)  38 (100.0)  39 (100.0)  39 (100.0)  37 (100.0)  39 (100.0)  38 (100.0)  36 (100.0) |
| Please describe any additional content that you consider useful within a pharmacological/pharmaceutical consultation:   - ‚Interactions with food.‘ - ‘Design of an individual medication plan together.’ - ‘Advising on individual medical treatment and illness – this is not possible when picking up medications from the pharmacy at home.’ | | | | |
| Do you consider a pharmacological/pharmaceutical consultation to be feasible as a telephone appointment or video conference?  Yes, definitely  Yes, partially  Neutral  Rather not  Not at all | **43 (100.0)**  9 (20.9)  11 (25.6)  10 (23.3)  7 (16.3)  6 (14.0) | | | |
| Do you consider it useful to provide written information material on oral drugs and their use at home?  Yes, definitely  Yes, partially  Neutral  Rather not  Not at all | **43 (100.0)**  16 (37.2)  23 (53.5)  2 (4.7)  2 (4.7)  - | | | |
| Which type of information material do you consider to be helpful?  Brochures about side effects  Drug fact sheets  Intake plans  Information brochures for safe handling (OAT) | **43 (100.0)**  34 (79.1)  33 (76.7)  27 (62.8)  24 (55.8) | | | |

S6 Table. Healthcare professionals.

| Survey question | Healthcare professionals (N=36) | | | |
| --- | --- | --- | --- | --- |
| **Oral medication education** | **No of responses (%)** | | | |
| Do you feel that you can provide your patients and their caregivers with sufficient information when prescribing a new oral drug?  Yes, definitely  Yes, partially  Neutral  Rather not  Not at all | **36 (100.0)**  2 (5.6)  17 (47.2)  11 (30.6)  6 (16.7)  - | | | |
| From your point of view, what are challenges with oral medication education?  Communication barriers  Lack of information material  Questions outside my expertise (e.g., pharmaceutical topics)  Lack of time  Lack of acceptance  Other | **36 (100.0)**  29 (80.6)  24 (66.7)  17 (47.2)  14 (38.9)  5 (13.9)  2 (5.6) | | | |
| Do you consider a pharmacological/pharmaceutical consultation on the use of oral drugs to be useful?  Yes, definitely  Yes, partially  Neutral  Rather not  Not at all | **36 (100.0)**  20 (55.6)  13 (36.1)  3 (8.3)  -  - | | | |
| Do you consider a pharmacological/pharmaceutical consultation on the use of OAT to be useful?  Yes, definitely  Yes, partially  Neutral  Rather not  Not at all | **36 (100.0)**  32 (88.9)  3 (8.3)  1 (2.8)  -  - | | | |
| If not, please describe why you do not consider a pharmacological/pharmaceutical consultation on the use of oral drugs to be useful:  - | | | | |
| At what point in the therapy do you consider a pharmacological/  pharmaceutical consultation on the use of oral drugs to be useful?  At time of hospital discharge  At time of new prescription of an oral drug  At time of first oncological diagnosis  Before a therapy block  As needed  Other | **36 (100.0)**  20 (55.6)  17 (47.2)  16 (44.4)  15 (41.7)  6 (16.7)  3 (8.3) | | | |
| How would you grade the importance of the following contents within a pharmacological/pharmaceutical consultation?  Prevention of side effects  Time of intake  Swallowing problems  Application instructions  Adherence  Handling of side effects  Interactions  Mode of action  Storage  Duration of intake  Complementary therapy  Procurement  Indication | Very important | Important | Less important | Total |
|  | 34 (94.4)  32 (88.9)  32 (88.9)  31 (86.1)  31 (86.1)  30 (83.3)  29 (80.6)  25 (69.4)  24 (66.7)  23 (63.9)  19 (52.8)  19 (52.8)  19 (52.8) | 1 (2.8)  4 (11.1)  2 (5.6)  5 (13.9)  2 (5.6)  5 (13.9)  5 (13.9)  10 (27.8)  12 (33.3)  9 (25.0)  9 (25.0)  13 (36.1)  10 (27.8) | 1 (2.8)  -  2 (5.6)  -  3 (8.3)  1 (2.8)  2 (5.6)  1 (2.8)  -  4 (11.1)  8 (22.2)  4 (11.1)  7 (19.4) | **36 (100.0)** |

| Please describe any additional content that you consider useful within a pharmacological/pharmaceutical consultation:   - ‘Discussion of advantages and disadvantages of different dosage forms.’ - ‚Interactions with food.‘ - ‘Procedure in case of forgetting the intake and information about different dosage forms.’ - ‘Procedure in case of vomiting after intake.’ - ‚Interactions with food.‘ - ‚Interactions with food.‘ - ‘Duration of intake (maximum allowed duration and dosage).’ - ‚Interactions with food.‘ | |
| --- | --- |
| Do you consider a pharmacological/pharmaceutical consultation as a telephone appointment or video conference to be feasible?  Yes, definitely  Yes, partially  Neutral  Rather not  Not at all | **36 (100.0)**  9 (25.0)  14 (38.9)  10 (27.8)  2 (5.6)  1 (2.8) |
| Do you consider it useful to provide written information material on oral drugs and how to use it at home?  Yes, definitely  Yes, partially  Neutral  Rather not  Not at all | **36 (100.0)**  12 (33.3)  16 (44.4)  4 (11.1)  4 (11.1)  - |
| Do you consider it useful to provide written information material on oral anticancer therapeutics and their use at home?  Yes, definitely  Yes, partially  Neutral  Rather not  Not at all | **36 (100.0)**  27 (75.0)  6 (16.7)  2 (5.6)  1 (2.8)  - |
| Which type of information material do you consider to be helpful?  Drug fact sheet  Information brochures for safe handling (OAT)  Intake plan  Brochures about side effects  Other | **35 (100.0)**  29 (82.9)  29 (82.9)  24 (68.6)  19 (54.3)  3 (8.6) |

- 1. Section C ‘Side effects’

S7 Table. Relevance of side effects according to patients, caregivers, and healthcare professionals.

| CTCAE Term | Patients | | | | | | Caregivers | | | | Healthcare professionals | | | | |
| --- | --- | --- | --- | --- | --- | --- | --- | --- | --- | --- | --- | --- | --- | --- | --- |
|  | **+** | | **+-** | | **-** | **Total** | **+** | **+-** | **-** | **Total** | **+** | **+-** | **-** | **Total** |  |
| Dry mouth | 2 (9.1) | 2 (9.1) | | 18 (81.8) | | 22 (100.0) | 4 (10.0) | 3  (7.5) | 33  (82.5) | 40  (100.0) | 10 (30.3) | 18 (54.5) | 5 (15.2) | 33 (100.0) |  |
| Difficulty swallowing | 3  (13.6) | 3 (13.6) | | 16 (72.7) | | 22 (100.0) | 8 (19.5) | 8  (19.5) | 25  (61.0) | 41  (100.0) | 25 (75.8) | 7 (21.2) | 1 (3.0) | 33 (100.0) |  |
| Mouth/throat sores | 7 (31.8) | 6 (27.3) | | 9 (40.9) | | 22 (100.0) | 14 (33.3) | 8 (19.0) | 20 (47.6) | 42 (100.0) | 33 (97.1) | 1 (2.9) | - | 34 (100.0) |  |
| Voice quality changes | 2 (9.1) | 2 (9.1) | | 18 (81.8) | | 22 (100.0) | 6  (14.6) | 2  (4.9) | 33 (80.5) | 41 (100.0) | 22 (74.7) | 9 (26.5) | 3 (8.8) | 34 (100.0) |  |
| Hoarseness | - | 1 (4.8) | | 20 (95.2) | | 21 (100.0) | 3 (7.3) | 3  (7.3) | 35 (85.4) | 41 (100.0) | 10 (29.4) | 14 (41.2) | 10 (29.4) | 34 (100.0) |  |
| Sore throat | - | 1 (4.8) | | 20 (95.2) | | 21 (100.0) | 3 (7.3) | 3  (7.3) | 35 (85.4) | 41 (100.0) | 10 (29.4) | 14 (41.2) | 10 (29.4) | 32 (100.0) |  |
| Taste changes | 9 (40.9) | 4 (18.2) | | 9 (40.9) | | 22 (100.0) | 20 (48.8) | 5 (12.2) | 16 (39.0) | 41 (100.0) | 23 (71.9) | 7 (21.9) | 2 (6.3) | 32 (100.0) |  |
| Decreased appetite | 8 (36.4) | 5 (22.7) | | 9 (40.9) | | 22 (100.0) | 18 (43.9) | 12 (29.3) | 11 (26.8) | 41 (100.0) | 23 (69.7) | 9 (27.3) | 1 (3.0) | 33 (100.0) |  |
| Nausea | 14 (63.6) | 5 (22.7) | | 3 (13.6) | | 22 (100.0) | 22 (52.4) | 7 (16.7) | 13 (31.0) | 42 (100.0) | 33 (97.1) | 1 (2.9) | - | 34 (100.0) |  |
| Vomiting | 12 (54.5) | 4 (18.2) | | 6 (27.3) | | 22 (100.0) | 15 (35.7) | 6 (14.3) | 21 (50.0) | 42 (100.0) | 33 (97.1) | 1 (2.9) | - | 34 (100.0) |  |
| Heartburn | 2 (9.1) | 5 (22.7) | | 15 (68.2) | | 22 (100.0) | 4 (9.5) | 5 (11.9) | 33 (78.6) | 42 (100.0) | 24 (70.6) | 8 (23.5) | 2 (5.9) | 34 (100.0) |  |
| Gas | 1 (4.5) | 3 (13.6) | | 18 (81.8) | | 22 (100.0) | 7 (16.7) | 7 (16.7) | 28 (66.7) | 42 (100.0) | 13 (38.2) | 17 (50.0) | 4 (11.8) | 34 (100.0) |  |
| Bloating | 1 (4.5) | 3 (13.6) | | 18 (81.8) | | 22 (100.0) | 7 (16.7) | 7 (16.7) | 28 (66.7) | 42 (100.0) | 13 (38.2) | 17 (50.0) | 4 (11.8) | 34 (100.0) |  |
| Hiccups | 1 (4.5) | 4 (18.2) | | 17 (77.3) | | 22 (100.0) | 1 (2.4) | 9 (22.0) | 31 (75.6) | 41 (100.0) | 5 (14.3) | 13 (37.1) | 17 (48.6) | 35 (100.0) |  |
| Constipation | 2 (9.1) | 5 (22.7) | | 15 (68.2) | | 22 (100.0) | 9 (22.5) | 8 (20.0) | 23 (57.5) | 40 (100.0) | 21 (61.8) | 13 (38.2) | - | 34 (100.0) |  |
| Diarrhea | 2 (9.1) | 4 (18.2) | | 16 (72.7) | | 22 (100.0) | 6 (14.3) | 8 (19.0) | 28 (66.7) | 42 (100.0) | 25 (73.5) | 9 (26.5) | - | 34 (100.0) |  |
| Abdominal pain | 2 (9.1) | 3 (13.6) | | 17 (77.3) | | 22 (100.0) | 4 (9.8) | 5 (12.2) | 32 (78.0) | 41 (100.0) | 17 (48.6) | 7 (20.0) | 11 (31.4) | 35 (100.0) |  |
| Fecal incontinence | - | - | | 22 (100.0) | | 22 (100.0) | 0 (0.0) | 5 (12.2) | 36 (87.8) | 41 (100.0) | 15 (44.1) | 6 (17.6) | 13 (38.2) | 34 (100.0) |  |
| Shortness of breath | - | 4 (19.0) | | 17 (80.1) | | 21 (100.0) | 3 (7.3) | 3 (7.3) | 35 (85.4) | 41 (100.0) | 20 (58.8) | 5 (14.7) | 9 (26.5) | 34 (100.0) |  |
| Cough | - | 2 (9.1) | | 20 (90.9) | | 22 (100.0) | 1 (2.4) | 6 (14.3) | 35 (83.3) | 42 (100.0) | 13 (38.2) | 14 (41.2) | 7 (20.6) | 34 (100.0) |  |
| Wheezing | - | 3 (13.6) | | 19 (86.4) | | 22 (100.0) | 1 (2.4) | 6 (14.6) | 34 (82.9) | 41 (100.0) | 14 (41.2) | 12 (35.9) | 8 (23.5) | 34 (100.0) |  |
| Sneezing | - | - | | 22 (100.0) | | 22 (100.0) | 1 (2.4) | 4 (9.5) | 37 (88.1) | 42 (100.0) | 7 (20.6) | 8 (23.5) | 19 (55.9) | 34 (100.0) |  |
| Swelling | 2 (9.1) | 2 (9.1) | | 18 (81.8) | | 22 (100.0) | 2 (4.9) | 2 (4.9) | 37 (90.2) | 41 (100.0) | 8 (23.5) | 17 (50.0) | 9 (26.5) | 34 (100.0) |  |
| Heart palpitations | 2 (9.1) | 3 (13.6) | | 17 (77.3) | | 22 (100.0) | 2 (4.8) | 11 (26.2) | 29 (69.0) | 42 (100.0) | 10 (30.3) | 17 (51.5) | 6 (18.2) | 33 (100.0) |  |
| Skin dryness | 7 (31.8) | 6 (27.3) | | 9 (40.9) | | 22 (100.0) | 17 (40.5) | 7 (16.7) | 18 (42.9) | 42 (100.0) | 8 (23.5) | 18 (52.9) | 8 (23.5) | 34 (100.0) |  |
| Acne | - | - | | 22 (100.0) | | 22 (100.0) | 2 (4.9) | 1 (2.4) | 38 (92.7) | 41 (100.0) | 16 (45.7) | 13 (37.1) | 6 (17.1) | 35 (100.0) |  |
| Hair loss^a^ | 21 (95.5) | - | | 1 (4.5) | | 22 (100.0) | 35 (83.3) | 2 (4.8) | 5 (11.9) | 42 (100.0) | 28 (82.4) | 5 (14.7) | 1 (2.9) | 34 (100.0) |  |
| Itching | 5 (22.7) | 4 (18.2) | | 13 (59.1) | | 22 (100.0) | 7 (16.7) | 11 (26.2) | 21 (50.0) | 42 (100.0) | 30 (88.2) | 4 (11.8) | - | 34 (100.0) |  |
| Hives | 1 (4.8) | - | | 20 (95.2) | | 21 (100.0) | 1 (2.5) | 3 (7.5) | 36 (90.0) | 40 (100.0) | 18 (52.9) | 5 (14.7) | 11 (32.4) | 34 (100.0) |  |
| Sensitivity to sunlight | 2 (9.1) | 3 (13.6) | | 17 (77.3) | | 22 (100.0) | 2 (4.9) | 8 (19.5) | 31 (75.6) | 41 (100.0) | 11 (33.3) | 14 (42.4) | 8 (24.2) | 33 (100.0) |  |
| Skin ulceration | - | 1 (4.5) | | 21 (95.5) | | 22 (100.0) | 5 (12.5) | 1 (2.5) | 34 (85.0) | 40 (100.0) | 22 (66.7) | 6 (18.2) | 5 (15.2) | 33 (100.0) |  |
| Numbness & tingling | 1 (4.5) | 2 (9.1) | | 19 (86.4) | | 22 (100.0) | 1 (2.4) | 8 (19.0) | 33 (78.6) | 42 (100.0) | 21 (63.6) | 10 (30.3) | 2 (6.1) | 33 (100.0) |  |
| Dizziness | 2 (9.1) | 2 (9.1) | | 18 (81.8) | | 22 (100.0) | 3 (7.1) | 7 (16.7) | 30 (71.4) | 42 (100.0) | 24 (75.0) | 6 (18.8) | 2 (6.3) | 32 (100.0) |  |
| Blurred vision | - | 2 (9.1) | | 20 (90.9) | | 22 (100.0) | 1 (2.4) | 4 (9.5) | 37 (88.1) | 42 (100.0) | 22 (66.7) | 6 (18.2) | 5 (15.2) | 33 (100.0) |  |
| Flashing lights | - | - | | 22 (100.0) | | 22 (100.0) | - | 1 (2.4) | 41 (97.6) | 42 (100.0) | 17 (51.5) | 5 (15.2) | 11 (33.3) | 33 (100.0) |  |
| Watery eyes | - | - | | 22 (100.0) | | 22 (100.0) | 2 (4.8) | 4 (9.5) | 36 (85.7) | 42 (100.0) | 11 (34.4) | 11 (34.4) | 10 (31.3) | 32 (100.0) |  |
| Ringing in ears | - | 3 (13.6) | | 19 (86.4) | | 22 (100.0) | 1 (2.4) | 4 (9.5) | 37 (88.1) | 42 (100.0) | 11 (33.3) | 10 (30.3) | 12 (36.4) | 33 (100.0) |  |
| Dry eyes | - | 1 (4.5) | | 21 (95.5) | | 22 (100.0) | 2 (4.8) | 8 (19.0) | 32 (76.2) | 42 (100.0) | 10 (30.3) | 16 (48.5) | 7 (21.2) | 33 (100.0) |  |
| Concentration | 5 (22.7 | 5 (22.7) | | 12 (54.5) | | 22 (100.0) | 4 (9.8) | 5 (12.2) | 28 (68.3) | 41 (100.0) | 12 (36.4) | 14 (42.4) | 7 (21.2) | 33 (100.0) |  |
| Memory | - | 1 (4.5) | | 21 (95.5) | | 22 (100.0) | 1 (2.4) | 4 (9.6) | 37 (88.1) | 42 (100.0) | 15 (45.5) | 9 (27.3) | 9 (27.3) | 33 (100.0) |  |
| General pain | 5 (22.7) | 7 (31.8) | | 10 (45.5) | | 22 (100.0) | 9 (21.4) | 15 (35.7) | 18 (42.9) | 42 (100.0) | 29 (87.9) | 4 (12.1) | - | 33 (100.0) |  |
| Headache | 5 (22.7) | 5 (22.7) | | 12 (45.5) | | 22 (100.0) | 6 (14.3) | 11 (26.2) | 25 (59.5) | 42 (100.0) | 30 (90.9) | 3 (9.1) | - | 33 (100.0) |  |
| Muscle pain | 3 (13.6) | 8 (36.4) | | 11 (50.0) | | 22 (100.0) | 8 (19.5) | 14 (34.1) | 19 (46.3) | 41 (100.0) | 22 (66.7) | 11 (33.3) | - | 33 (100.0) |  |
| Joint pain | 3 (13.6) | 5 (22.7) | | 14 (63.6) | | 22 (100.0) | 8 (18.0) | 15 (35.7) | 19 (45.2) | 42 (100.0) | 19 (57.6) | 13 (39.4) | 1 (3.0) | 33 (100.0) |  |
| Insomnia | 5 (22.7) | 3 (13.6) | | 14 (63.6) | | 22 (100.0) | 5 (12.5) | 8 (20.0) | 27 (67.5) | 40 (100.0) | 23 (71.9) | 6 (18.8) | 3 (9.4) | 32 (100.0) |  |
| Fatigue | 8 (36.4) | 6 (27.3) | | 8 (36.4) | | 22 (100.0) | 16 (38.1) | 20 (47.6) | 6 (14.3) | 42 (100.0) | 27 (81.8) | 6 (18.2) | - | 33 (100.0) |  |
| Anxious | 6 (27.3) | 1 (4.5) | | 15 (68.2) | | 22 (100.0) | 11 (26.8) | 8 (19.5) | 22 (53.7) | 41 (100.0) | 28 (84.8) | 3 (9.1) | 2 (6.1) | 33 (100.0) |  |
| Sad | 6 (27.3) | 6 (27.3) | | 10 (45.5) | | 22 (100.0) | 8 (19.0) | 10 (23.8) | 24 (57.1) | 42 (100.0) | 23 (69.7) | 7 (21.2) | 3 (9.1) | 33 (100.0) |  |
| Suicidal ideation | - | - | | 22 (100.0) | | 22 (100.0) | - | - | 42 (100.0) | 42 (100.0) | 14 (43.8) | 6 (18.8) | 12 (37.5) | 32 (100.0) |  |
| Painful urination | - | 1 (4.5) | | 21 (95.5) | | 22 (100.0) | - | 3 (7.1) | 39 (92.9) | 42 (100.0) | 22 (66.7) | 6 (18.2) | 5 (15.2) | 33 (100.0) |  |
| Urinary urgency | 1 (4.5) | 3 (13.6) | | 18 (81.8) | | 22 (100.0) | 3 (7.5) | 3 (7.5) | 34 (85.0) | 40 (100.0) | 13 (39.4) | 13 (39.4) | 7 (21.2) | 33 (100.0) |  |
| Urinary frequency | - | - | | 20 (100.0) | | 20 (100.0) | 3 (7.5) | 7 (17.5) | 30 (75.0) | 40 (100.0) | 13 (39.4) | 14 (42.4) | 6 (18.2) | 33 (100.0) |  |
| Change in usual urine color | 1 (4.8) | 5 (23.8) | | 15 (71.4) | | 21 (100.0) | 5 (11.9) | 7 (16.7) | 33 (78.6) | 42 (100.0) | - | 10 (30.3) | 23 (69.7) | 33 (100.0) |  |
| Urinary incontinence | - | - | | 22 (100.0) | | 22 (100.0) | 1 (2.5) | 3 (7.5) | 36 (90.0) | 40 (100.0) | 14 (42.4) | 13 (39.4) | 6 (18.2) | 33 (100.0) |  |
| Bruising | - | 4 (18.2) | | 18 (81.8) | | 22 (100.0) | - | 9 (22.0) | 32 (78.0) | 41 (100.0) | 6 (18.2) | 12 (36.4) | 15 (45.5) | 33 (100.0) |  |
| Chills | 3 (13.6) | 2 (9.1) | | 17 (77.3) | | 22 (100.0) | 4 (10.0) | 6 (15.0) | 30 (75.0) | 40 (100.0) | 18 (54.5) | 11 (33.3) | 4 (12.1) | 33 (100.0) |  |
| Increased sweating | 5 (22.7) | 4 (18.2) | | 13 (59.1) | | 22 (100.0) | 6 (14.3) | 6 (14.3) | 30 (71.4) | 42 (100.0) | 12 (36.4) | 16 (48.5) | 5 (15.2) | 33 (100.0) |  |
| Hot flashes | 3 (13.6) | 2 (9.1) | | 17 (22.3) | | 22 (100.0) | 5 (11.9) | 4 (9.5) | 33 (78.6) | 42 (100.0) | 10 (30.3) | 17 (51.5) | 6 (18.2) | 33 (100.0) |  |
| Nosebleed | 2 (9.1) | 1 (4.5) | | 19 (86.4) | | 22 (100.0) | 3 (7.1) | 3 (7.1) | 36 (85.7) | 42 (100.0) | 15 (45.5) | 14 (42.4) | 4 (12.1) | 33 (100.0) |  |
| Falls | - | 4 (18.2) | | 18 (81.8) | | 22 (100.0) | 3 (7.1) | 7 (16.7) | 32 (76.2) | 42 (100.0) | 3 (9.4) | 16 (50.0) | 8 (25.0) | 32 (100.0) |  |
| Muscle weakness | 6 (27.3) | 3 (13.6) | | 13 (59.1) | | 22 (100.0) | 8 (19.0) | 8 (19.0) | 26 (61.9) | 42 (100.0) | 18 (54.5) | 12 (36.4) | 3 (9.1) | 33 (100.0) |  |
| Restlessness | 3 (13.6) | 4 (18.2) | | 15 (68.2) | | 22 (100.0) | 8 (19.0) | 9 (21.4) | 25 (59.5) | 42 (100.0) | 15 (45.5) | 12 (36.4) | 5 (15.2) | 33 (100.0) |  |

relevant (+); neutral (+-); less relevant (-); green: side effect terms drawn from the Ped-Pro-CTCAE^®^ graded as being relevant by at least 20% of participants of all three groups; grey: core CTCAE terms published by Reeve et al. [2], ^a^excluded as no prophylactic or relieving measures are possible

S8 Table. Caregivers.

| Survey question | Caregivers (N= 44) |
| --- | --- |
| **Side effects** | **No of responses (%)** |
| Do you feel you can easily recognise side effects of your child’s oncological treatment?  Yes, definitely  Yes, partially  Neutral  Rather not  Not at all | **43 (100.0)**  11 (25.6)  24 (55.8)  7 (16.3)  1 (2.3)  - |
| Do you feel you can easily recognise the severity of side effects of your child oncological treatment?  Yes, definitely  Yes, partially  Neutral  Rather not  Not at all | **43 (100.0)**  8 (18.6)  21 (48.8)  11 (25.6)  3 (7.0)  - |

S9 Table. Healthcare professionals.

| Survey question | Healthcare professionals (N= 36) |
| --- | --- |
| **Side effects** | **No of responses (%)** |
| Do you feel you can easily recognise side effects of your patients‘ oncological treatment?  Yes, definitely  Yes, partially  Neutral  Rather not  Not at all | **35 (100.0)**  5 (14.3)  23 (65.7)  7 (20.0)  -  - |
| Do you feel you can easily recognise the severity of side effects of your patients‘ oncological treatment?  Yes, definitely  Yes, partially  Neutral  Rather not  Not at all | **35 (100.0)**  7 (20.0)  21 (60.0)  5 (14.3)  2 (5.7)  - |
| Do you consider a self-monitoring of side effects for children to be useful?  Yes, definitely  Yes, partially  Neutral  Rather not  Not at all | **35 (100.0)**  4 (11.4)  19 (54.3)  8 (22.9)  3 (8.6)  1 (2.9) |
| I feel confident that self-monitoring of side effects helps to detect serious side effects more quickly.  Strongly agree  Agree  Neutral  Disagree  Strongly disagree | **35 (100.0)**  8 (22.9)  16 (45.7)  9 (25.7)  2 (5.7)  - |
| I feel confident that self-monitoring of side effects facilitate their handling.  Strongly agree  Agree  Neutral  Disagree  Strongly disagree | **35 (100.0)**  10 (28.6)  13 (37.1)  7 (20.0)  5 (14.3)  - |
| I worry that self-monitoring of side effects leads to preventable stress for patients and caregivers.  Strongly agree  Agree  Neutral  Disagree  Strongly disagree | **35 (100.0)**  1 (2.9)  9 (25.7)  9 (25.7)  12 (34.3)  4 (11.4) |
| I worry that self-monitoring of side-effects might favor their occurence.  Strongly agree  Agree  Neutral  Disagree  Strongly disagree | **35 (100.0)**  3 (8.6)  9 (25.7)  12 (34.3)  8 (22.9)  3 (8.6) |
| I worry that self-monitoring of side-effects might lead to more frequent hospitalisation.  Strongly agree  Agree  Neutral  Disagree  Strongly disagree | **35 (100.0)**  -  6 (17.1)  13 (37.1)  12 (34.3)  4 (11.4) |

1. Adaptation of the AMBORA intervention

Details on the new youngAMBORA information material compared to the AMBORA trial [2] are shown in Table S10. Number and times of consultation sessions and clinical parameters to be evaluated at the respective time are presented in Fig. 5. The satisfaction with the care program will be measured with the help of a self-designed questionnaire in week 2 or week 4.

*Key element: Counselling and training*

Counselling sessions will be assisted by self-designed youngAMBORA information material:

- Drug fact sheets with the following paediatric-specific information added to existing AMBORA drug fact sheets:
  - Potential off-label use
  - Dosages for children (including potential different dosages on different days based on body surface area)
  - Swallowing problems and reference to workbook
  - Side effects occurring predominantly in children
  - Safety aspects for caregivers
  - Vaccinations to avoid during treatment
  - Application instructions for suspensions (if suspensions are commercially available)
  - Alternate dosage formulations (if valid information is available in the literature)
- A brochure for safe handling of OAT in the home setting, containing information about proper storage, disposal, handling of body fluids, and drug spills.
- Illustrated information brochures presenting mode of actions of the most commonly prescribed drug groups (antimetabolites, protein kinase inhibitors, alkylating agents, topoisomerase inhibitors).

As an example, the drug fact sheet for the most commonly prescribed OAT ‘mercaptopurine’ is shown in Figure S1.

In case of discontinuous intake for at least two weeks (patient group II), patients and their caregivers will be consulted at time of OAT prescription (week 0), week 1 and week 2. In case of continuous OAT intake for at least 12 weeks (patient group I), counselling sessions will be conducted in week 0, week 1, week 4, and week 12 (Fig. 6).

Caregivers’/patients’ knowledge about newly prescribed OAT will be measured using the validated SIMS-D [3] questionnaire.

*Key element: Side effect prevention and management*

Brochures about the most relevant identified side effects were designed, including child-friendly information about their prevention and handling. Additionally, patient-tailored video clips about the occurrence of side effects and the most common ones (*‘Mouth/throat sores’, ‘Nausea and vomiting’, ‘Diarrhea’,* and *‘Constipation’*) were created. Incidence and severity of all 17 relevant side effects will be systematically recorded from patients’ (from the age of 6 years) and caregivers’ point of view using the Ped-PRO-CTCAE^®^ [4] questionnaire. Patients’ health-related quality of life at predefined times will be recorded with the cancer module of the validated PedsQL™ questionnaire [5,6].

*Key element: Adherence counselling*

An interactive, child-friendly workbook for swallowing drugs was designed to facilitate drug application and to optimise drug adherence. Intake plans from the AMBORA trial were adapted to paediatrics by including the opportunity of using stamps or stickers to reward a successful intake. Drug adherence will be recorded using the MARS-D [7] questionnaire.

*Key element: Medication management*

Structured medication analyses will be performed at any counselling session. Medications errors will be categorised using two valid classification systems: ‘Pharmaceutical Care Network Europe’ (PCNE V9.1) [8] to assess their cause and ‘National Coordinating Council for Medication Error Reporting and Prevention’ (NCC MERP) [9] to define their severity. If required, individual medication plans will be provided.

S1 Fig. Drug fact sheet – Mercaptopurine.


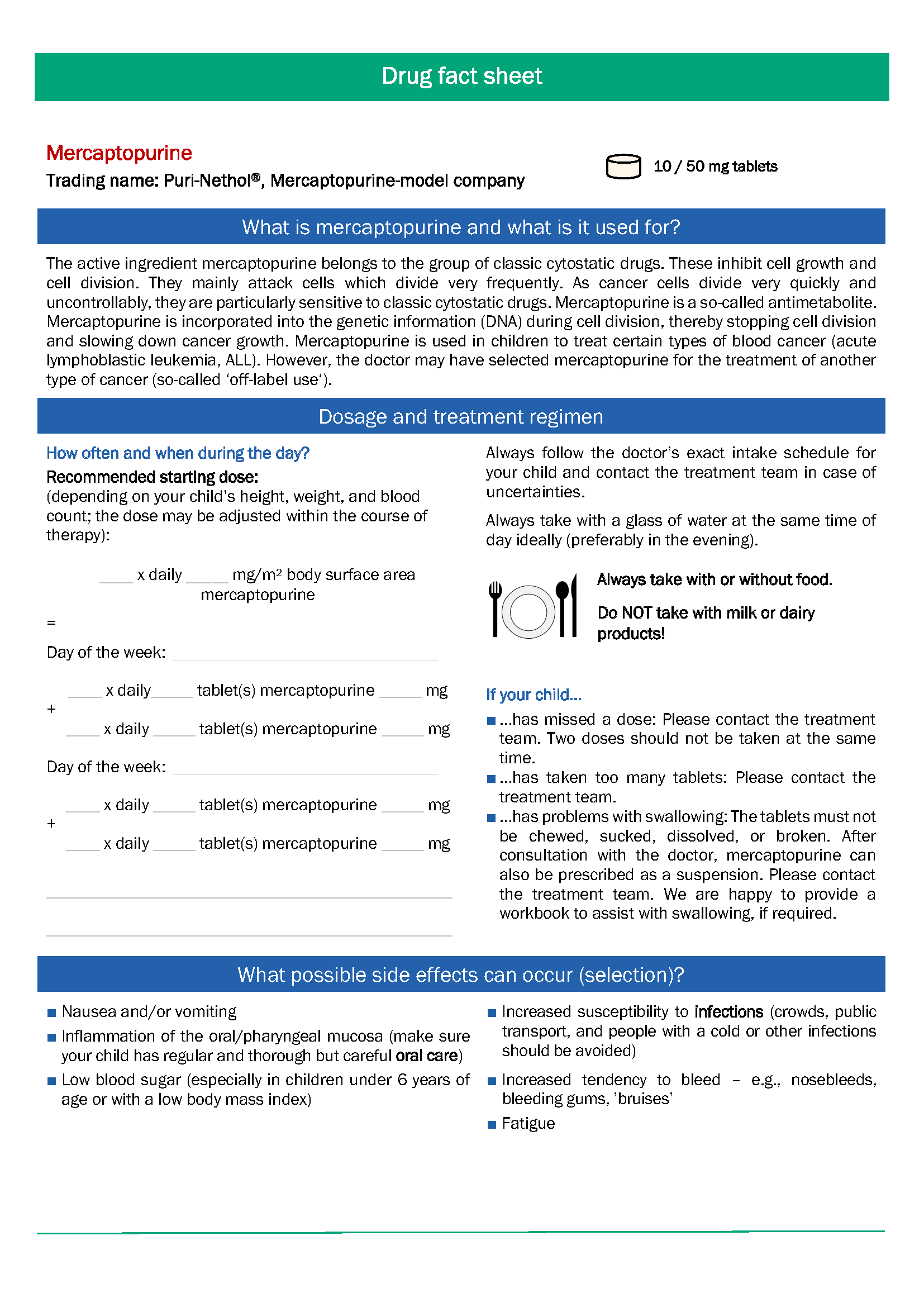


S10 Table. Overview of the provided information material in the AMBORA trial [1] and in the youngAMBORA care program.

| **Key elements** | **AMBORA trial [2]** | **youngAMBORA** | **Number** |
| --- | --- | --- | --- |
| Counselling and training | OAT fact sheets | OAT fact sheets | 25 |
|  |  | Information brochure for safe handling | 1 |
|  |  | Information brochures on the mode of action | 4 |
|  |  | Information brochures for alternate dosage formulation | 4 |
| Side effect prevention and management | Information brochures | Information brochures | 9 |
|  |  | Patient-tailored video clips | 4 |
| Adherence counselling | Drug intake plans | Drug intake plans | 8 |
|  |  | Workbook for swallowing drugs | 1 |
| Medication management | Individual medication plan | | - |


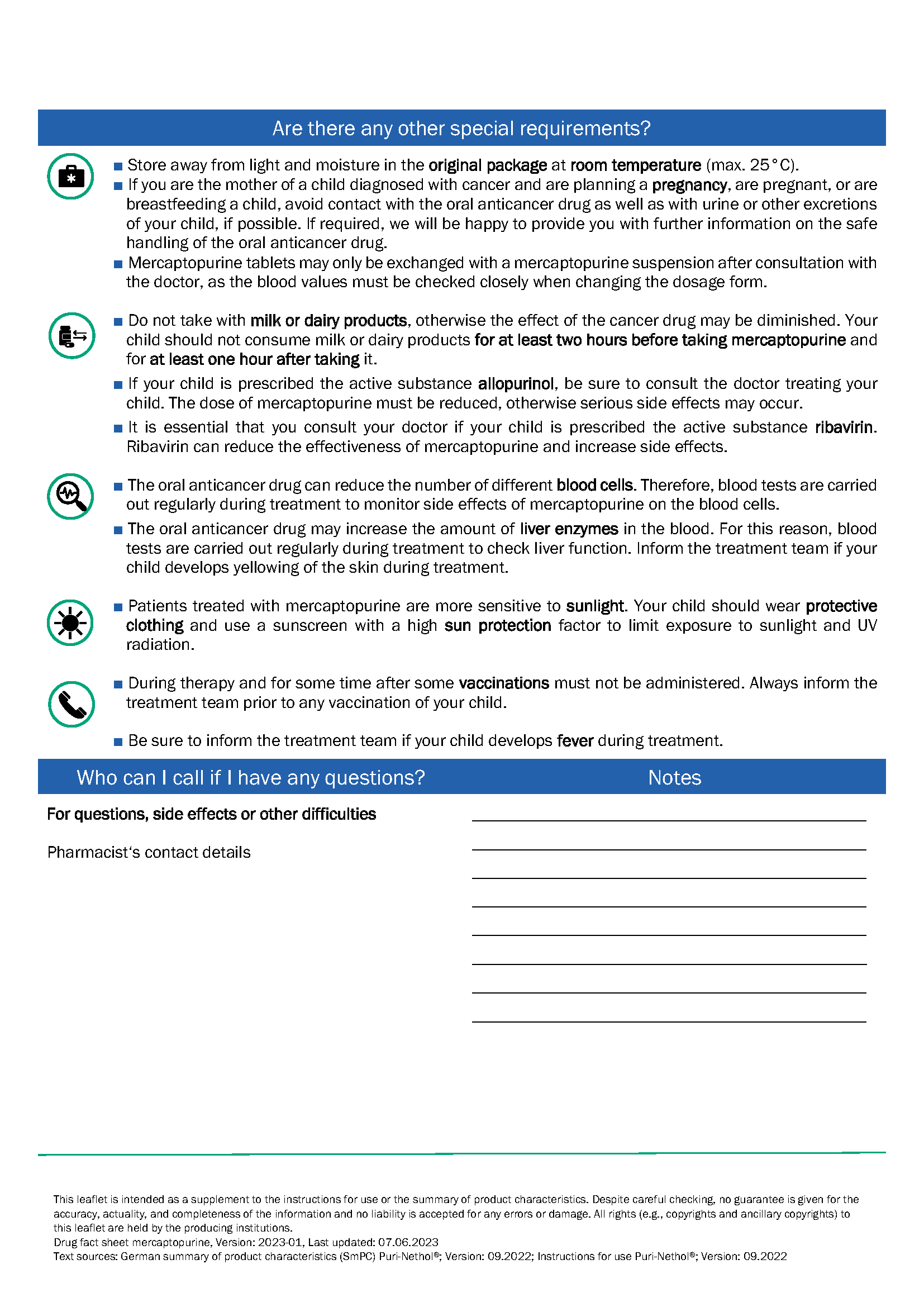


Designed information material

S11 Table. OAT fact sheets.

| Cyclophosphamide tablets | Mercaptopurine tablets |
| --- | --- |
| Dabrafenib capsules | Methotrexate tablets |
| Dabrafenib capsules + trametinib tablets | Selumetinib capsules |
| Dasatinib suspension | Tazemetostat tablets |
| Dasatinib tablets | Temozolomide capsules* |
| Etoposide capsules* | Thalidomide capsules |
| Hydroxycarbamide capsules | Tioguanine tablets |
| Idarubicine capsules | Topotecan capsules* |
| Imatinib tablets for suspension | Trametinib tablets |
| Imatinib capsules and tablets* | Trofosfamide tablets |
| Lenvatinib capsules* | Venetoclax tablets |
| Lomustine capsules | Vinorelbine capsules |
| Mercaptopurine suspension |  |

* Including information brochures for alternate dosage formulation

S12 Table. Information brochures about side effect prevention and management.

| Decreased appetite and taste changes |  |
| --- | --- |
| Diarrhea | |
| Fatigue | |
| Side effects of steroids | |
| Skin dryness/skin changes | |
| Insomnia | |
| Mouth/throat sores | |
| Nausea and vomiting | |
| Constipation | |

1. References

1. Dürr P, Schlichtig K, Kelz C, Deutsch B, Maas R, Eckart MJ, et al. The randomized AMBORA trial: Impact of pharmacological/pharmaceutical care on medication safety and patient-reported outcomes during treatment with new oral anticancer agents. J Clin Oncol. 2021;39(18):1983-1994.

2. Reeve BB, Withycombe JS, Baker JN, Hooke MC, Lyons JC, Mowbray C, et al. The first step to integrating the child's voice in adverse event reporting in oncology trials: A content validation study among pediatric oncology clinicians. Pediatr Blood Cancer. 2013;60(7):1231-1236.

3. Mahler C, Jank S, Hermann K, Horne R, Ludt S, Haefeli WE, et al. Psychometric properties of a german version of the "Satisfaction with Information about Medicines Scale" (SIMS-D). Value Health. 2009;12(8):1176-1179.

4. National Cancer Institute: Pediatric module of Patient Reported Outcomes version of the Common Terminology Criteria for Adverse Events (Ped-PRO-CTCAE^®^) measurement system, item library 2023 [cited 2024 January 15]. Available from: <https://healthcaredelivery.cancer.gov/pro-ctcae/instrument-ped.html>.

5. Varni JW, Burwinkle TM, Katz ER, Meeske K, Dickinson P. The PedsQL in pediatric cancer: Reliability and validity of the Pediatric Quality of Life Inventory generic core scales, multidimensional fatigue scale, and cancer module. Cancer. 2002;94(7):2090-2106.

6. Robert RS, Paxton RJ, Palla SL, Yang G, Askins MA, Joy SE, et al. Feasibility, reliability, and validity of the Pediatric Quality of Life Inventory ™ generic core scales, cancer module, and multidimensional fatigue scale in long-term adult survivors of pediatric cancer. Pediatr Blood Cancer. 2012;59(4):703-707.

7. Mahler C, Hermann K, Horne R, Ludt S, Haefeli WE, Szecsenyi J, et al. Assessing reported adherence to pharmacological treatment recommendations. Translation and evaluation of the Medication Adherence Report Scale (MARS) in Germany. J Eval Clin Pract. 2010;16(3):574-579.

8. Pharmaceutical Care Network Europe Association. Classification for drug related problems, V9.1. 2003-2020 [cited 2024 February 5]. Available from: <https://www.pcne.org/upload/files/417_PCNE_classification_V9-1_final.pdf>.

9. Snyder RA, Abarca J, Meza JL, Rothschild JM, Rizos A, Bates DW. Reliability evaluation of the adapted national coordinating council medication error reporting and prevention (NCC MERP) index. Pharmacoepidemiol Drug Saf. 2007;16(9):1006-1013.
