# Supplementary material for: Medication safety with oral antitumour therapeutics in paediatrics (youngAMBORA): A mixed-methods approach towards a tailored care program
Source: PLoS One. 2024 Dec 5;19(12):e0315077. doi: 10.1371/journal.pone.0315077 (PMC11620591; doi:10.1371/journal.pone.0315077)
Supplement: Attachment — Submitted filename: ResponseToReviewers.docx [file pone.0315077.s002.docx]

**Point-by-Point Response to the Editor’s and Reviewers’ Comments**

***(The Editor’s and Reviewers’ comments are shown in bold italics)***

We thank all Editors and Reviewers for their very valuable and important comments! We addressed all remarks in the responses given below.

All modifications throughout the manuscript are marked in the track-change version. The line numbers refer to the track-change version of the modified manuscript and the revised clean version, respectively (e.g., track-change version: page. 2, line 69; clean version: p. 2, l. 45).

Furthermore, we adjusted minor inconsistencies, that we noticed during our in-depth review (e.g., revised Figure 4). However, no content-related changes were made and all respective modifications are marked in the track-change mode.

**Comments from the Editor:**

***Please ensure that your manuscript meets PLOS ONE's style requirements, including those for file naming. The PLOS ONE style templates can be found at*** [***https://journals.plos.org/plosone/s/file?id=wjVg/PLOSOne_formatting_sample_main_body.pdf***](https://journals.plos.org/plosone/s/file?id=wjVg/PLOSOne_formatting_sample_main_body.pdf) ***and*** [***https://journals.plos.org/plosone/s/file?id=ba62/PLOSOne_formatting_sample_title_authors_affiliations.pdf***](https://journals.plos.org/plosone/s/file?id=ba62/PLOSOne_formatting_sample_title_authors_affiliations.pdf)

Response: Thank you for this information! We again checked and verified that our manuscript meets all PLOS ONE’s style requirements.

***In the online submission form, you indicated that all relevant data are within the manuscript and its Supporting information file. Additional individual, pseudonymized data is available upon request. All PLOS journals now require all data underlying the findings described in their manuscript to be freely available to other researchers, either 1. In a public repository, 2. Within the manuscript itself, or 3. Uploaded as supplementary information. This policy applies to all data except where public deposition would breach compliance with the protocol approved by your research ethics board. If your data cannot be made publicly available for ethical or legal reasons (e.g., public availability would compromise patient privacy), please explain your reasons on resubmission and your exemption request will be escalated for approval.***

Response: As indicated, all relevant data are already included in the manuscript and its supporting information file. No additional individual, pseudonymised data is available upon request. Please consider these changes concerning our data availability statement.

***Your ethics statement should only appear in the Methods section of your manuscript. If your ethics statement is written in any section besides the Methods, please move it to the Methods section and delete it from any other section. Please ensure that your ethics statement is included in your manuscript, as the ethics statement entered into the online submission form will not be published alongside your manuscript.***

Response: Thank you for this remark. As suggested, we moved the ethics statement and the note on waiving informed consent to the Methods section (track-change: p. 9, l. 179-185; clean: p. 9, l. 163-169). We also updated the waiver number (23-273_1-ANF) provided by the Ethics Committee of the Friedrich-Alexander-Universität Erlangen-Nürnberg, which was uploaded as an English translation during the submission process (track-change: p. 9, l. 183; clean: p. 9, l. 167)

**Reviewers’ comments**

**Reviewer 1:**

1. ***The sequence of events is not well timed. There is a mismatch between the collected retrospective data and questionnaire-based survey. There is sheer disconnect between sequence of events. The authors need to clarify why there is a time difference in that***

Response: Thank you for raising this important aspect! We purposely chose different time periods for the following reasons:

For the quantitative, retrospective analyses, we aimed to use the maximum available data to obtain representative results by compensating the low incidence rates in paediatrics with a longer data collection period. We used the most recent and reliably accessible outpatient and inpatient data from our University paediatric cancer centre to analyse the number of oncological entities and oral antitumor therapeutics (OAT) prescriptions. To improve clarity, we slightly expanded the Methods section (track-change: p. 7, l. 114; clean: p. 6, l. 108).

For the qualitative part, we performed a prospective questionnaire-based survey. Previous studies showed, that in qualitative research reliable results can be achieved with small sample sizes of 9 to 17 [1]. Therefore, we considered a survey of 22 participating patients, 44 caregivers, and 36 healthcare professionals appropriate. Furthermore, we observed similar results within an interim analysis and could conclude that higher participation rates in the questionnaires did not lead to a change in results, which is why we stopped the survey after seven months. We included this aspect in the Methods section (track-change: p. 7, l. 132-133; clean: p. 7, l. 118-119). A time period of seven months would have been too short for quantitative statements in the retrospective analyses. In view of these considerations, the advantages of our approach outweigh the disadvantages.

In general, finding appropriate time intervals for data collection is a challenge associated with mixed-methods research. We therefore added this important point to the Discussion section (track-change: p. 20-21, l. 430-434; clean: p. 20, l. 411-415).

**Reviewer 2:**

# 1. *The study is well designed and analyzed, however, the presentation and flow of the study information could be simpler and less confusing. Authors must highlight how the study results can be clinically applicable.*

Response: We appreciate the positive feedback on our work! We tried to make the flow of the study information more understandable by rearranging the Methods section and including an explanatory paragraph at the beginning, referring to Figure 1, which describes the structure of the manuscript (track-change: p. 6, l. 88-100; clean: p. 6, l. 88-99). Furthermore, we used the respective subheadings shown in Figure 1 consistently throughout the manuscript.

We now emphasised the clinical applicability of our results within the Discussion section [‘Identified challenges can be directly addressed in the tailored youngAMBORA care program to improve OAT handling, treatment adherence, self-management of side effects, and ultimately patient outcomes. To the best of our knowledge, similar concepts to the youngAMBORA care program including a comprehensive and continuous support of paediatric patients treated with OAT are lacking. In general, clinical pharmacologist/pharmacist services in outpatient units are not common practice within the German healthcare setting. Therefore, tailored support can lead to increased treatment safety and effectiveness, especially in the outpatient setting, which is known for a high risk of drug-related problems’ (track-change: p. 20, l. 408-417; clean: p. 19-20, l. 389-398)] and would like to further refer to the detailed explanations and changes made under point 9.

# 2. *The inclusion criteria says--if they/their child were either receiving at least one oral drug at the time of data collection or had been treated with OAT or any other oral drug in the past and if an oncological diagnosis was more than three months ago. --do the authors imply non-Oncological oral drugs also being assessed here? If that the case then the study results cannot be applied to only OAT!*

Response: Indeed, we also assessed the intake of any other, non-oncological drugs in the survey. This is an important point that we did not describe clearly enough so far. So far, this aspect has just been mentioned briefly in the Methods Section (track-change: p. 8, l. 138-141; clean: p. 7, l. 124-127) and in Table 1 (track-change: p. 12, l. 235-238; clean: p. 12, l. 219-222). We now specified this aspect in the Methods section (track-change: p. 8, l. 139; clean: p. 7, l. 125).

As you correctly indicated in the next point, paediatric oncology practice mainly involves parenteral chemotherapy. For this reason, we extended the inclusion criteria to all oral drugs to achieve higher response rates. However, the majority of participating patients and caregivers were already experienced in OAT handling at the time of the survey (Table 1 and track-change: p. 20, l. 422-423; clean: p. 20, l. 403-404). Beyond that, described challenges - such as application difficulties - do not only affect OAT but also other oral drugs, which is why we did not identify any differences in the responses.

Additionally, advanced medication reviews are carried out within every youngAMBORA consultation session, addressing problems related to non-oncological oral drugs (e.g., supportive therapy) as well.

We therefore assume a good transferability of our survey results to the developed care program – only including patients taking OAT. Nevertheless, we agree that this aspect should be further stressed as a limitation of our work and expanded our respective statement in the Discussion section (track-change: p. 20, l. 419-420; clean: p. 20, l. 400-401).

# 3. *Pediatric Oncology practice mainly involves parenteral chemo [except ALL/Ly] in the First line setting. When we use oral drugs, it is may times in the palliative chemo setting. Can authors clarify, the treatment setting for these patients. Authors should also discuss whether this could change patient/caregiver response.*

Response: As you correctly stated, OAT are most frequently used in the palliative chemo setting with off-label use being very common. In the developed youngAMBORA care program, patients and caregivers are consulted regardless of whether the treatment intention is curative or palliative. To address this important aspect, we added two explanatory statements in the Introduction and Discussion section (track-change: p. 4, l. 66; clean: p. 4, l. 66 and track-change: p. 17-18, l. 355-357; clean: p. 17, l. 336-338).

In general, we consider the impact on the qualitative survey results to be of minor relevance due to the following reasons: The palliative treatment setting, on the one hand, is often perceived as the most demanding and individual situation. On the other hand, it is also characterised by higher healthcare capacities allowing a closer interaction with the patients and caregivers. Both aspects could potentially influence the survey results slightly in two different directions, but without affecting the overall conclusions.

Nevertheless, we discussed these important aspects of patients’/caregivers’ and healthcare professionals’ interactions in the palliative treatment setting in the Discussion section (track-change: p. 17, l. 350-353; clean: p. 17, l. 331-334).

# 4. *Reliability of survey data from Kids as young as 6 years is questionable as we all know, understanding of kids can be diverse and they can be influenced easily. Kids can have inconsistence responses depending on "surroundings and incentives!" I feel if Authors can redo study by just including kids order than 8 or 9 OR have some metric of assessing kids' understanding before enrolling in the study.*

Response: Thank you very much for these suggestions! In our daily clinical routine, we observe that the children’s behavior is very individual and depends a lot on the family situation and not just on their age. Due to these personal differences in development, it is generally very difficult to define a specific age at which children are able to complete questionnaires in a meaningful way.

Our rationale behind the decision to include patients aged 6 years and older was the fact that other questionnaires commonly used in paediatrics (e.g., Peds-QL™ [2,3]) are validated for this patient age. We added this important information to the Methods section (track-change: p. 8 l. 141-142; clean: p. 7, l. 127-128). Beyond that, excluding patients under the age of 8 or 9 would have led to an underestimation of the problems mainly occurring in this younger age group and therefore to a limited transferability of our results. As the overall number of patients per age group was too small to draw conclusions, why we refrained from retrospectively narrowing down the patient collective on the basis of age. Nevertheless, we added the precise age distribution of the surveyed patients to the revised Table 1 (track-change: p. 12 l. 235-238; clean: p. 12, l. 219-222), which additionally shows that the absolute number of participating patients aged 8 years or younger was relatively low (36.4%; 8/22).

Taken together, it is unfortunately not possible to redo the study, as the survey was conducted prior to the implementation of the youngAMBORA care program. Currently, all patients receive a pharmacological/pharmaceutical consultation at the initiation of an oral antitumour drug and would be biased when answering the questionnaire.

# 5. *One of the survey questions for patients "Do you find it difficult to remember to take oral drugs regularly "--almost all times kids are given meds by parents/caregiver [except may be adolescents] so question does not apply to whole patient group.*

Response: We agree with you – thank you for stressing this aspect! As described above, our experience has shown that there are very large individual and age-independent differences in patient behavior and in the question of who takes responsibility for the regular medication intake. Regardless of the patient’s age, we therefore encourage the families to take responsibility together.

With the mentioned question, our aim was to sensitise the children to the important topic of adherence. Moreover, we wanted the questionnaire to be uniform for the entire surveyed group, which is why we did not differentiate between different age groups.

As shown in the revised Table 1 (track-change: p. 12 l. 235-238; clean: p. 12, l. 219-222), the proportion of surveyed children being 8 years or younger was lower than the proportion of older children (36.4%; 8/22 vs. 63.6%; 14/22). Additionally, the following table shows that there were no differences in the responses for the various age groups (Table I). We therefore refrained from adding the detailed information included in Table I to the Supporting Information.

Table I. Answers according to age groups.

| Survey question | Patients (N=22) |
| --- | --- |
| **Administration and handling of oral drugs** | **No of responses (%)** |
| **Age of patient 6-8 years** | |
| Do you find it difficult to remember to take oral drugs regularly (adherence)?  Yes, I find it difficult  Sometimes yes, sometimes no  No, I don’t find it difficult | **7 (100.0)**  1 (14.3)  3 (42.9)  3 (42.9) |
| **Age of patient 9-12 years** | |
| Do you find it difficult to remember to take oral drugs regularly (adherence)?  Yes, I find it difficult  Sometimes yes, sometimes no  No, I don’t find it difficult | **7 (100.0)**  1 (14.3)  3 (42.9)  3 (42.9) |
| **Age of patient 13-18 years** | |
| Do you find it difficult to remember to take oral drugs regularly (adherence)?  Yes, I find it difficult  Sometimes yes, sometimes no  No, I don’t find it difficult | **7 (100.0)**  2 (28.6)  1 (14.3)  4 (57.1) |

# 6. *Kids undergoing cancer therapy commonly have NG/OG tube and the authors have not separated concerns with NG tube administration vs oral administration. I hope they can exclude the patients on getting drug via NG approach or NG+oral approach. For example, Taste/smell is not an issue with NG approach.*

Response: As the survey data was collected anonymously, we unfortunately are not able to analyse the use of feeding tubes retrospectively. Generally, in our University paediatric cancer centre, the use of NG/OG tubes is not very common. Nevertheless, we are currently evaluating this aspect in the youngAMBORA care program, as we are aware of the importance of this issue. The unpublished, preliminary data of 47 patients counseled in the youngAMBORA care program so far, showed that a minority of only 2 patients had a feeding tube (i.e., a PEG tube) at OAT initiation. To be even more specific, the drug was administered orally and not by the tube in both cases. We therefore consider this aspect to be of minor relevance within the survey, but will analyse the use of feeding tubes in the youngAMBORA patients.

# 7. *Some of the survey questionnaire is difficult to comprehend--For example-- Does your child have difficulties swallowing tablets or capsules at time of data collection? Yes, definitely Yes, partially Neutral Rather not Not at all What is the interpretation of "rather not" in response? Response choices should be like Yes, always Yes. most times Yes, occasionally No, never. there are other questions with similar confusing answer choices. Example, Did your child refuse to take oral drugs in the past? Yes, definitely Yes, partially Neutral Rather not Not at all*

# Response: Thank you for mentioning this aspect, which we also considered at the beginning of the study. As briefly mentioned in the Methods section, all three versions of questionnaires were peer-reviewed by two representatives of each group in order to assess comprehensibility prior to their use (track-change: p. 8, l. 147-150; clean: p. 7, l. 131-134).

# We decided to use a 5-point Likert scale, as the patient version with three simple answer options (green, grey, and red smiley) also contained a neutral possibility (grey smiley). We specified our rationale within the revised version of the manuscript by adding information in the Methods section (track-change: p. 7-8, l. 135-137; clean: p. 7, l. 121-123). A retrospective change to a 4-point Likert scale would distinctively bias the results and is not feasible. We therefore refrained from changing the scale in the presented survey but will take your suggestion into account for the development of future questionnaires.

# 8. *The authors frequently refer AMBORA trial data in the discussion/result section. This could be trimmed and does not add to the study findings. Other authors can compare how adult responses differ vs Pediatric.*

# Response: We thank you for the suggestion to trim the references to the AMBORA trial data, which we have implemented in the Results and Discussion section. In particular, we used the wording ‘AMBORA trial’ less frequently (track-change: p. 10, l. 207-208; clean: p. 10, l. 191; track-change: p. 11, l. 214; clean: p. 11, l. 197-198 and track-change: p. 15, l. 328; clean: p. 16, l. 312) and shortened the interpretation of tumour entities observed in the AMBORA trial cohort (track-change: p. 17, l. 339-341; clean: p. 17, l. 322).

# 9. *How the study findings can be used to improve patient and disease related outcomes needs to be highlighted.*

# Response:

In general, the development of tailored care programs for children should be based on the assessment of child-specific needs in the respective targeted group, as described by Preston et al. [4]. Previous work in other projects showed that pre-evaluation of caregivers’ needs led to the successful establishment of tailored paediatric palliative care programs, which have a high impact on patient-related outcomes in real-world nowadays [5, 6]. In order to discuss the relevance of our work in a broader context of literature, we added a respective statement and references number 41 and 42 in the Discussion section (track-change: p. 19-20, l. 407-408; clean: p. 19, l. 388-389).

# In detail, our present work provides the basis for a tailored care program for paediatric patients treated with OAT by 1) identifying relevant CTCAE terms to be focused on and 2) by developing appropriate information material (information brochures about side effect prevention and management and age-appropriate video clips for children). As one of four key elements within the youngAMBORA care program, structured side effect management could lead to the early detection or even prevention of severe side effects that are known to be a risk factor for treatment discontinuation [7]. Furthermore, the qualitative survey enabled us to identify important application problems, which we can now address more precisely in the youngAMBORA care program. As shown in other studies [8], an improved handling of OAT in the home setting can lead to increased treatment safety and effectiveness.

# The possible benefit of the youngAMBORA care program on clinical outcomes (e.g., medication errors and patient-reported outcomes) is currently being investigated, but we consider its impact to be high. To highlight the potential patient and disease-related outcomes of our work, we amended the Discussion section in the manuscript accordingly (track-change: p. 20, l. 408-417; clean: p. 19-20, l. 389-398).

# 10. *How youngAMBORA care program differ from periodic pharmacist review practiced at most hospitals? For example, it is common practice in the west to have monthly pharmacist assessment with parents/patient about chemo adherence/administration etc.*

# Response: Thank you for this comment! In contrast to periodic pharmacist reviews, the youngAMBORA care program includes continuous and direct contact with patients and their caregivers, as shortly mentioned in the first version of the manuscript (track-change: p. 14, l. 299-301; clean: p. 14, l. 283-285). Personal consultations take place at defined time points, but patients and/or caregivers can also contact us in person or by telephone at any time with questions or complaints on a low-threshold basis. Beyond that, the structured intervention of the youngAMBORA care program is unique and consists of the four key elements: counselling and training, side effect prevention and management, adherence counselling, and medication management. To the best of our knowledge, there is no other pharmacology/pharmacy service providing tailored information material to a similar extent. We added a brief statement in the Discussion section to provide more clarity (track-change: p. 20, l. 411-413; clean: p. 20, l. 392-394).

# While it may be routine in other countries, it is not common practice that patients and caregivers are consulted by a clinical pharmacologist/pharmacist immediately at the time of OAT initiation in Germany. As another innovative aspect, it should be noted, that the youngAMBORA care program is mainly offered in the outpatient setting, while clinical pharmacologist/pharmacist services are currently predominantly provided in the inpatient sector within the German healthcare setting [9]. Outpatient treatment in particular is prone to application problems and medication errors underlining the importance of intensified support [10]. As also mentioned by the caregivers in the qualitative survey (S5 Table), handling of OAT-related problems is challenging and might not be sufficiently addressed by any retail pharmacy. We included a statement and a new reference (43) in the Discussion section to emphasise the advantage of outpatient care programs (track-change: p. 20, l. 415-417; clean: p. 20, l. 394-398).

# 11. *The side effects are naturally vague and can be grouped as it is confusion and adds to the study jargon. For example, Muscle pain, General pain, Fatigue can be grouped. Similarly urinary side effects*

# Response: As mentioned in the Methods section (track-change: p. 8, l. 156-159; clean: p. 8, l. 140-143), we based our work on the paediatric module of the patient-reported outcomes version of the Common Terminology Criteria of Adverse Events (Ped-PRO-CTCAE^®^) [11] and included 62 side effects in the survey. We share your opinion that this selection is very detailed and might be confusing, which is why we set out to simplify the side effect evaluation to the 17 most relevant terms according to the results of our survey. However, we respectfully refrained from grouping the side effect terms in Figure 4D to remain consistent with the well-established Ped-PRO-CTCAE^®^ [11] questionnaire.

# 12. *Authors should highlight limitations of mixed-method research in the discussion section.*

# Response: Thank you for this constructive feedback, which we now further emphasised in the Discussion section. We included the following paragraph, which also addresses another Reviewer’s comment that we considered different time intervals in the quantitative and qualitative analyses as a valid limitation (see: track-change: p. 20-21, l. 428-434; clean: p. 20, l. 409-415):

# ‘The participation of more motivated patients of different ages from six onwards, caregivers, or healthcare professionals might have led to biased results of the qualitative analyses. In general, mixed-methods research is associated with the challenge of comparing and combining quantitative and qualitative data. For instance, we used the most recent and reliably accessible data for the quantitative analyses, but data interpretation might be limited by considering a different time interval in the qualitative evaluation.’.

# References

1. Hennink M, Kaiser BN. Sample sizes for saturation in qualitative research: A systematic review of empirical tests. Soc Sci Med. 2022;292:114523.
2. Robert RS, Paxton RJ, Palla SL, Yang G, Askins MA, Joy SE, et al. Feasibility, reliability, and validity of the Pediatric Quality of Life Inventory ™ generic core scales, cancer module, and multidimensional fatigue scale in long-term adult survivors of pediatric cancer. Pediatr Blood Cancer. 2012;59(4):703-707.
3. Varni JW, Burwinkle TM, Katz ER, Meeske K, Dickinson P. The PedsQL in pediatric cancer: Reliability and validity of the Pediatric Quality of Life Inventory generic core scales, multidimensional fatigue scale, and cancer module. Cancer. 2002;94(7):2090-2106.
4. Preston J, Nafria B, Ohmer A, Gaillard S, Dicks P, West L, et al. Developing a more tailored approach to patient and public involvement with children and families in pediatric clinical research: Lessons learned. Ther Innov Regul Sci. 2022;56(6):948-963.
5. Verberne LM, Kars MC, Schouten-van Meeteren AYN, van den Bergh EMM, Bosman DK, Colenbrander DA, et al. Parental experiences and coping strategies when caring for a child receiving paediatric palliative care: a qualitative study. Eur J Pediatr. 2019;178(7):1075-1085.
6. Spraker-Perlman HL, Aglio T, Kaye EC, Levine D, Barnett B, Berry Carter K, et al. Leveraging grief: Involving bereaved parents in pediatric palliative oncology program planning and development. Children (Basel). 2021;8(6).
7. Dürr P, Schlichtig K, Kelz C, Deutsch B, Maas R, Eckart MJ, et al. The randomized AMBORA trial: Impact of pharmacological/pharmaceutical care on medication safety and patient-reported outcomes during treatment with new oral anticancer agents. J Clin Oncol. 2021;39(18):1983-1994.
8. Akkawi El Edelbi R, Eksborg S, Ekman J, Lindemalm S. Improved home management of oral pediatric anticancer drugs as a result of an intervention comprising practical training, written instructions and movie clips: A pilot study. J Oncol Pharm Pract. 2023;29(4):818-825.
9. Schulz C, Fischer A, Vogt W, Leichenberg K, Warnke U, Liekweg A, et al. Clinical pharmacy services in Germany: A national survey. Eur J Hosp Pharm. 2021;28(6):301-305.
10. Naseralallah L, Stewart D, Price M, Paudyal V. Prevalence, contributing factors, and interventions to reduce medication errors in outpatient and ambulatory settings: A systematic review. Int J Clin Pharm. 2023;45(6):1359-1377.
11. National Cancer Institute: Pediatric module of Patient Reported Outcomes version of the Common Terminology Criteria for Adverse Events (Ped-PRO-CTCAE^®^) measurement system, item library 2023 [cited 2024 January 15]. Available from: https://healthcaredelivery.cancer.gov/pro-ctcae/.
